# Plasticity of Adult Human Pancreatic Duct Cells by Neurogenin3-Mediated Reprogramming

**DOI:** 10.1371/journal.pone.0037055

**Published:** 2012-05-14

**Authors:** Nathalie Swales, Geert A. Martens, Stefan Bonné, Yves Heremans, Rehannah Borup, Mark Van de Casteele, Zhidong Ling, Daniel Pipeleers, Philippe Ravassard, Finn Nielsen, Jorge Ferrer, Harry Heimberg

**Affiliations:** 1 Diabetes Research Center, Vrije Universiteit Brussel, Brussels, Belgium; 2 Microarray Facility, Rigshospitalet, Copenhagen, Denmark; 3 Centre de Recherche Institut du Cerveau et de la Moelle, CNRS UMR7225, Université Pierre et Marie Curie, Paris, France; 4 Genomic Programming of Beta Cells Laboratory, Institut d’Investigacions Biomèdiques August Pi i Sunyer, Barcelona, Spain; VIB & Katholieke Universiteit Leuven, Belgium

## Abstract

**Aims/Hypothesis:**

Duct cells isolated from adult human pancreas can be reprogrammed to express islet beta cell genes by adenoviral transduction of the developmental transcription factor neurogenin3 (Ngn3). In this study we aimed to fully characterize the extent of this reprogramming and intended to improve it.

**Methods:**

The extent of the Ngn3-mediated duct-to-endocrine cell reprogramming was measured employing genome wide mRNA profiling. By modulation of the Delta-Notch signaling or addition of pancreatic endocrine transcription factors Myt1, MafA and Pdx1 we intended to improve the reprogramming.

**Results:**

Ngn3 stimulates duct cells to express a focused set of genes that are characteristic for islet endocrine cells and/or neural tissues. This neuro-endocrine shift however, is incomplete with less than 10% of full duct-to-endocrine reprogramming achieved. Transduction of exogenous Ngn3 activates endogenous Ngn3 suggesting auto-activation of this gene. Furthermore, pancreatic endocrine reprogramming of human duct cells can be moderately enhanced by inhibition of Delta-Notch signaling as well as by co-expressing the transcription factor Myt1, but not MafA and Pdx1.

**Conclusions/Interpretation:**

The results provide further insight into the plasticity of adult human duct cells and suggest measurable routes to enhance Ngn3-mediated *in vitro* reprogramming protocols for regenerative beta cell therapy in diabetes.

## Introduction

In the budding ducts of the embryonic pancreas, neurogenin3 (Ngn3) activation initiates the differentiation of epithelial progenitors to islet endocrine cells [1], [2], [3]. The presence of insulin-producing cells in or close to the ductal epithelium of adult human pancreas [4] has prompted the idea that islet endocrine cells in the adult pancreas could bud off the ducts in the same way as they are generated during embryogenesis. This process that is referred to as beta cell neogenesis remains hypothetical in the human organ since cell lineage tracing or specific protein markers for the newly formed cells are lacking. Is the potential for endocrine reprogramming restricted to specific cell populations within the ducts? Can this process be recapitulated in vitro and will it be possible to improve its efficiency in vitro to yield clinically useful grafts for beta cell transplantation? The answer to the latter questions was partly given by previous finding that *in vitro*, human duct cells hold the potential for partial endocrine reprogramming [5], [6], [7], [8], [9]. Previously, we demonstrated that ectopic expression of mouse Ngn3 in cytokeratin 19 (CK19) positive human duct cell cultures induced expression of several endocrine transcripts such as synaptophysin (Syp), prohormone convertase 1/3, glucokinase and insulin (Ins) [6].

The objectives of the present study were twofold. First, we aimed to fully characterize the extent of Ngn3-mediated duct-to-endocrine cell reprogramming, employing genome wide mRNA profiling. Secondly, based on the newly gained knowledge we intended to improve the reprogramming efficiency by enhancing Ngn3-effects on adult human duct cells through (i) modulation of Delta-Notch signaling [6], [10] or (ii) addition of pancreatic endocrine transcription factors myelin transcription factor 1 (Myt1) [11], [12], [13], MafA and Pdx1 [14]. We report that while Ngn3 reprograms duct cells to a neuro-endocrine phenotype, only a minor part of the estimated roadmap that needs to be followed to fully convert exocrine duct to endocrine islet cells is achieved by Ngn3 alone. In this model mouse Ngn3 interacts with the promoter of human Ngn3 leading to its gene activation. Enhancing this interaction by suppression of Delta-Notch signaling is insufficient to further drive the reprogramming. While co-expression of Ngn3 with MafA+Pdx1 also fails to drive the reprogramming, the combination of Ngn3 and Myt1 is more promising.

## Results

### Exogenous Ngn3 Activates Both Neuronal and Endocrine Traits in Adult Human Duct Cells

Adult human duct cells were transduced with adenoviruses expressing Ngn3 and GFP (AdGFP-Ngn3) or GFP only (AdGFP) and cultured in suspension for 3 or 14 days to study their genome-wide mRNA expression. Tables S1 and S2 list all transcripts that were either 1.5-fold (LB) up- or down regulated in Ngn3-transduced duct cells at day 3 (68 up, 36 down), day 14 (105 up, 19 down) or both. The Ngn3-associated transcriptome shift consisted of 188 different genes that were altered at least at one of the 2 time points tested (140 up and 48 down). Ngn3-activated genes were however statistically (P<0.001) enriched in gene ontologies relating to development, neurogenesis, synaptic transmission, regulated hormone secretion and nuclear regulators. Genes belonging to these overrepresented clusters are shown in Table 1. Conventional and real-time PCR confirmed activation of genes coding for beta cell transcription factors (*NeuroD1*, *Pax4*, *Nkx2.2*), vesicle proteins (*Scg3, Syp*) insulin processing (*Pcsk1*) and nutrient sensing (*Gck*), but also genes that are considered characteristic for neural cells but are present in beta cells as well (*Pcp4, Rab26*) (Figure S1).

**Table 1:** : Selection of Ngn3-activated genes in suspension cultured human duct cells.

Gene Symbol	Accession	Gene name	Fold Ngn3/Ctrl				Fold Ngn3		endocrine	Cluster
			D3		D14		D14/D3		abundance	Fig. 2
**Development - Neurogenesis**										
ISL1	NM_002202	ISL1 transcription factor, LIM/homeodomain, (islet-1)	1,2		2,4	*	2,4	*	26,9	A
INSM1	NM_002196	insulinoma-associated 1	64,7	*	82,4	*	3,3	*	25,3	A
NKX2-2	NM_002509	NK2 transcription factor related, locus 2 (Drosophila)	5,4	*	23,3	*	6,3	*	19,9	A
Ascl1	BC001638	achaete-scute complex-like 1 (Drosophila)	1,1		48,9	*	54,9	*	4,4	A
BACE1	NM_012104	beta-site APP-cleaving enzyme 1	1,2		1,9	*	1,5		2	A
NEUROD1	NM_002500	neurogenic differentiation 1	4,2	*	30,9	*	12,5	*	21	B
NLGN1	NM_014932	neuroligin 1	−1,5		1		2,1	*	1,4	B
SIM1	AL121948	single-minded homolog 1 (Drosophila)	1,2		1,4		2	*	1,2	B
PAX4	AB008913	paired box gene 4	1,5		2,4	*	2	*	1	B
OLFML2B	AW007573	olfactomedin-like 2B	3,6	*	2		−1,5		0,7	B
NEUROG3	NM_020999	neurogenin 3	1,7		12,5	*	5,9	*	0,7	B
NHLH1	M96739	nescient helix loop helix 1	1,2		2,3	*	1,9		0,6	B
NEUROG1	NM_006161	neurogenin 1	2,7	*	1,1		−2,2		0,5	B
NHLH2	AA166895	nescient helix loop helix 2	1,6		4,3	*	3	*	0,5	B
STMN2	BF967657	stathmin-like 2	1,5		5,5	*	7,4	*	10,8	C
PCP4	NM_006198	Purkinje cell protein 4	2,1		4,7	*	3,4	*	5,9	C
APLP1	U48437	amyloid beta (A4) precursor-like protein 1	1,6		3,5		5,8	*	5,8	C
TM4SF2	NM_004615	tetraspanin 7/transmembrane 4 superfamily member 2	1,3		2,5	*	2,7	*	5	C
SERPINI1	NM_005025	serine (or cysteine) proteinase inhibitor, clade I, member 1	2		2,6	*	−1,2		2,1	C
AF1Q	BC006471	ALL1-fused gene from chromosome 1 q	1		1,4		1,9	*	1,2	C
NPTX1	NM_002522	neuronal pentraxin I	45,3	*	64,1		1,3		0,9	C
MT3	NM_005954	metallothionein 3 (growth inhibitory factor (neurotrophic))	2,1	*	1,2		−1,6		0,6	C
S100A1	NM_006271	S100 calcium binding protein A 1	5,4	*	9,2	*	2		0,5	C
PAX6	AW088232	paired box gene 6 (aniridia, keratitis)	1,2		5,3	*	5,2	*	−	−
FOXA2	AL121722	hepatocyte nuclear factor 3, beta	2,4	*	2,6	*	−1,3		−	−
PHC2	AA431100	polyhomeotic-like 2 (Drosophila)	−1,2		1,2		2	*	−	−
GDAP1	BF002104	ganglioside-induced differentiation-associated protein 1	1,2		2,9	*	2		−	−
**Neuroendocrine - Synaptic Function**										
SGNE1	NM_003020	secretory granule, neuroendocrine protein 1 (7B2 protein)	1,1		2,8	*	2,9	*	62,5	A
SCG3	NM_013243	secretogranin III	3,9		7,5	*	5,1	*	55,6	A
SCG2	NM_003469	secretogranin II (chromogranin C)	1,3		6,9	*	6,5	*	31,1	A
CPE	NM_001873	carboxypeptidase E	1,7		3	*	2,2	*	29,2	A
SST	NM_001048	somatostatin	−1,1		2,5		5,8	*	28,4	A
CHGB	NM_001819	chromogranin B (secretogranin 1)	1,5		2,7	*	3,1	*	27	A
PCSK1	NM_000439	proprotein convertase subtilisin/kexin type 1	1,4		19,7	*	15,9	*	17,3	A
PCSK2	NM_002594	proprotein convertase subtilisin/kexin type 2	1,8		2,4		3,3	*	15,4	A
CHGA	NM_001275	chromogranin A (parathyroid secretory protein 1)	1,4		10,4	*	12,2	*	14,2	A
ENPP2	L35594	ectonucleotide pyrophosphatase/phosphodiesterase 2	1,6		5,3	*	3,8	*	13	A
SCGN	NM_006998	secretagogin, EF -hand calcium binding protein	3,2		23,8	*	7,6	*	9,8	A
KCNH2	NM_000238	potassium voltage-gated channel, subfamily H, member 2	−1		2,4	*	2,2		4	A
CADPS	NM_003716	Ca2+-dependent secretion activator	1,4		2,4	*	1,5		6,2	B
NBEA	NM_015678	neurobeachin	1,1		2,1	*	1,7		1,4	B
RAB26	NM_014353	RAB26, member RAS oncogene family	3,6	*	8,4	*	2,6		1	B
RTN2	NM_005619	reticulon 2	7,9	*	13,4		−1,8		0,7	B
HPCA	BC001777	hippocalcin	8,1		8,8	*	7,2	*	0,6	B
SNAP25	NM_003081	synaptosomal-associated protein, 25 kDa	−1		1,7		3,3	*	14,3	C
SCN1B	NM_001037	sodium channel, voltage-gated, type I, beta	1,5		2,5	*	1,9	*	2,4	C
SV2A	NM_014849	synaptic vesicle glycoprotein 2A	1,1		1,9		2,4	*	1,6	C
SYT11	AA626780	synaptotagmin XI	1,1		1,6		2,1	*	1,6	C
SYP	U93305	synaptophysin	1,1		3,6	*	2,8	*	1,4	C
RPIP8	AI197870	RaP 2 interacting protein 8	−1		1,5		1,8	*	1,2	C
SYNGR3	NM_004209	synaptogyrin 3	−1,4		1,1		1,9	*	1,2	C
CAMK2B	AF078803	calcium/calmodulin-dependent protein kinase II beta	1,2		2,4	*	2,1	*	1,1	C
KCNF1	AF029780	potassium voltage-gated channel, subfamily F, member 1	6,1		6,4	*	1,2		0,8	C
CABP7	AI989906	calcium binding protein 7	4,1	*	18,1	*	3,1	*	−	−
CPLX1	BC002471	complexin 1	3,7	*	3,5	*	1,1		−	−
CPNE5	AB046819	copine V	−3,2		−1,3		2,6	*	−	−
SYT4	AB037763	synaptotagmin IV	1		2,8	*	2,2	*	−	−
SYT13	AB037848	synaptotagmin XIII	1,1		2,1	*	1,7		−	−
Nuclear - Transcription										
ST18	NM_014682	suppression of tumorigenicity 18 (breast carcinoma)	1,4		7,9	*	4,9	*	11,4	A
ETV1	BE881590	ets variant gene 1	−1,5		1,6		2,2	*	9,6	A
BTBD3	NM_014962	BTB (POZ) domain containing 3	1		2	*	2	*	7,5	A
BEX1	NM_018476	brain expressed, X-linked 1	−1,2		1,6		2,2	*	7,4	A
NALP1	NM_021730	NACHT, leucine rich repeat and P Y D containing 1	1,1		10,3	*	9,9	*	7	A
CUL3	NM_003590	cullin 3	1		7,3	*	6,9	*	3,6	A
ZNF505	AC007204	zinc finger protein 505	2,9	*	1,4		−1,8		1,1	B
HMGB3	NM_005342	high-mobility group box 3	1,5		2,1	*	1,4		0,9	B
FEV	NM_017521	FEV (ETS oncogene family)	−1		9,3	*	12,7	*	0,8	B
LOC51337	NM_016647	mesenchymal stem cell protein DSCD75	1,1		2,1	*	1,8		0,7	B
BPY2	NM_004678	basic charge, Y-linked, 2	4	*	1,8		−2,2		0,5	B
SOX2	AI669815	SRY (sex determining region Y )-box 2	2,3		15	*	10,4	*	−	−
HIC2	AK024950	Hypermethylated in cancer 2	−1,1		1,5		3,8	*	−	−

**Figure 1:**
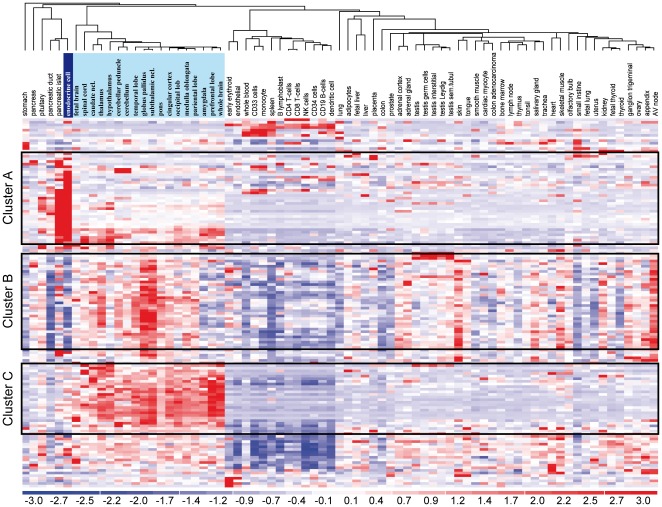
Ngn3 induces endocrine cell-enriched and neural-enriched genes in ducts. We analyzed all transcripts that are Ngn3-activated in suspension cultured duct cells (n = 140 on HG133A) in a public dataset of more than 50 human tissues and cell types (each n = 2), supplemented by our endocrine cell hybridizations (n = 3). The cluster graph thus shows the relative tissue-selectivity of the Ngn3-induced transcripts. Approximately 25% of Ngn3-regulated transcripts are selectively enriched in endocrine cells/pancreatic islets (cluster A). Cluster B genes are enriched in the central nervous system but generally not in pancreatic endocrine cells. Cluster C exhibit a neuro-endocrine expression pattern, with comparable expression in central nervous system and endocrine cells. An annotated version of Figure 1 with full gene names is included as Figure S2.

140 transcripts on HG133A array transcripts are at least 1.5-fold (LB) activated by AdGFP-Ngn3 in human duct cells. Ngn3-activated genes are statistically enriched (P<0.001) in gene ontologies of development, neurogenesis, synaptic transmission, regulated hormone secretion and nuclear regulators; genes belonging to these clusters are shown. From left to right, columns indicate fold transcript accumulation in AdGFP-Ngn3 versus AdGFP control cells at 3 (D3) or 14 (D14) days post transduction. Two columns on the right refer to the analysis of tissue tropism in Figure 1: endocrine cell abundance indicates relative mRNA abundance in human endocrine cells as compared to the other tissues profiled in Figure 1. Last column indicates in which cluster (A, B, C) of Figure 1 the gene is situated.

* P<0.05, n = 3.

The cell type-specificity of all genes that were activated by Ngn3 in human duct cells was further analyzed by integrating our expression profiles of untransduced human duct and beta cells with the Human Gene Atlas, containing more than 50 primary human tissues and cell types [15]. Hierarchical clustering (Figure 1) revealed that ± 25% of the Ngn3-activated genes in duct cells show an endocrine cell-specific expression (Figure 1, cluster A), ± 25% are more characteristic for neural tissues (Figure 1, cluster B) and another set of ± 20% transcripts appear similarly enriched in neurons and endocrine cells (Figure 1, cluster C). A fully annotated version of this heat map is provided as Figure S2. Combined, this indicates that Ngn3 activates a shift towards a neuro-endocrine phenotype.

### Exogenous Ngn3 alone Cannot Fully Reprogram Adult Human Pancreas Duct Cell Preparations into Islet Endocrine Cells

**Figure 2:**
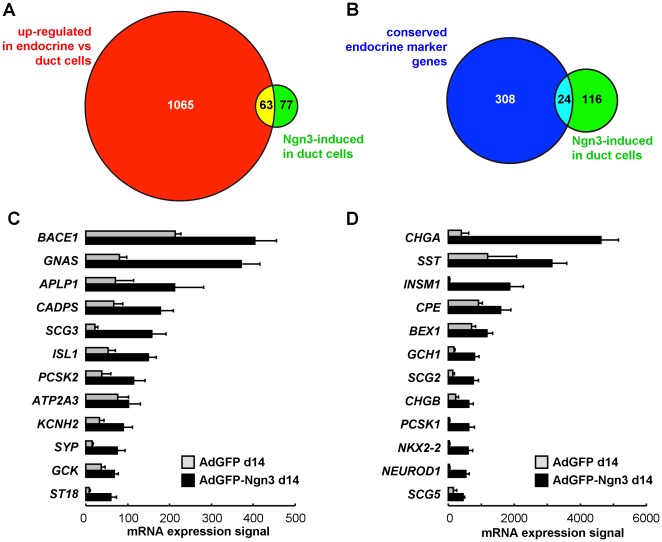
Extent of Ngn3-mediated duct-to-islet endocrine reprogramming. Transcripts that are ≥1.5-fold (LB) higher expressed in untransduced human endocrine versus suspension cultured duct cells (n = 1128 on the HG133A array, panel A, green) were compared to the 140 transcripts that were AdGFP-Ngn3-activated as compared to AdGFP-transduced duct cells using the same statistical criteria (panel A and B, gray). Nearly half (45%, 63 of 140) of Ngn3-regulated transcripts (panel A, intersect) are expressed in endocrine cells. Panel B compares these 140 Ngn3-activated transcripts (gray) to a previously reported [16] panel of 332 genes with beta cell-selective expression in mouse, rat and human. Ngn3 activated 24 of these endocrine marker genes. Gene chip mRNA expression signal (mean±SD, n = 3) in transduced duct cells is shown in panels C and D.

We estimated the extent of duct-to-endocrine reprogramming by two methods. First, in a genome-wide comparison, 1128 transcripts show 1.5-fold (LB, P<0.01) higher expression in untransduced human islets versus duct cells. This gene set thus needs to be activated for full conversion of a duct-to-endocrine enriched cell fraction, and together it can be used to estimate the required duct-to-beta reprogramming. Almost half of Ngn3-activated genes (63 of 140, Figure 2A) belong to this gene set, but overall Ngn3 fails to achieve full reprogramming with only 6% (63 of 1128 transcripts) of the estimated path completed. Second, Ngn3-activation focused on islet endocrine marker genes was analyzed (Figure 2B), using a previously reported panel of 332 genes with conserved beta cell-abundant expression [16] : Ngn3 activated 24 such marker genes in duct cells, reaching on the average 33% (range 6–104%) of the mRNA signal measured in human beta cell fractions (Figure 2C–D). This confirmed that reprogramming was incomplete, with 91% of conserved beta cell marker genes not Ngn3-regulated. Both approaches thus indicate that Ngn3 activates a restricted set of neural- and/or endocrine-specific genes reflecting clear initiation but not completion of pancreatic endocrine cell differentiation.

### Exogenous Mouse Ngn3 Binds the Promoter of the Endogenous Human Ngn3 gene and Provides Positive Feedback

**Figure 3:**
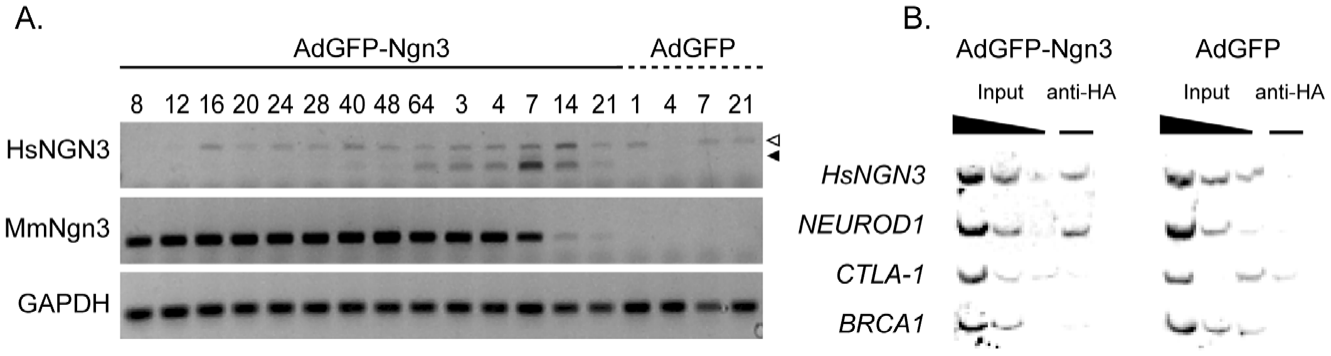
Ngn3 induces its own expression via direct binding on its own promoter. (A) RT-PCR detection of exogenous and endogenous Ngn3 in AdGFP-Ngn3 transduced duct cells. Time course detection of exogenous HA-Ngn3 and endogenous Ngn3 mRNA showing sustained endogenous Ngn3 expression. Time points are indicated in hours (8–64) or days (1–21). Open triangle: genomic Ngn3 PCR fragment; black triangle: endogenous Ngn3 cDNA PCR fragment. Gapdh-specific RT-PCR is included as template control (n = 3). (B) Chromatin immunoprecipitation with an anti-HA antibody on chromatin derived from adult human duct cells transduced with either AdGFP-Ngn3 or AdGFP. DNA from input chromatin was serially diluted as a reference for semiquantitative PCR analysis. In the AdGFP-Ngn3 samples both NGN3 and NEUROD1 promoter fragments are enriched with antibodies directed against the HA tag, suggesting that Ngn3 binds both endogenous wild type promoters. NGN3 and NEUROD1 promoter fragments were not detected in AdGFP control samples, neither were the negative control genomic fragments from the CTLA-1 and BRCA1 genes (n = 2).

Microarray profiling revealed a 12-fold up-regulation of the endogenous human neurogenin3 transcript (HsNgn3) in duct cells 14 days after transduction with cDNA encoding mouse neurogenin3 (MmNgn3) (Table 1). This is surprising since Ngn3 was reported to control its own expression through negative feedback during mouse embryonic development [17]. Because the vector-derived sequence is limited to the open reading frame of MmNgn3 and 11 out of the 12 HsNgn3-specific microarray probes are specific for the 3′ untranslated Ngn3 region, this represents genuine activation of endogenous HsNgn3. Increased expression of HsNgn3 was confirmed by RT-PCR primers that specifically amplify transcripts of either HA-tagged exogenous MmNgn3 cDNA or endogenous HsNgn3 (Figure 3A): the HsNgn3 transcript became detectable 2 days after AdGFP-Ngn3 transduction, peaking at 7 days and then decreased to reach the lower detection limit 3 weeks after transduction. Chromatin immunoprecipitation revealed an *in vivo* binding of HA-tagged MmNgn3 protein to the 5′ region of the HsNgn3 gene but not to negative control genomic fragments of the CTLA-1 and BRCA1 genes (Figure 3B). These studies therefore provide for the first time evidence for endogenous autoactivation of Ngn3.

### Delta-Notch Signaling Antagonizes Ngn3-induced HsNGN3 Activation and Neuro-endocrine Differentiation

**Figure 4:**
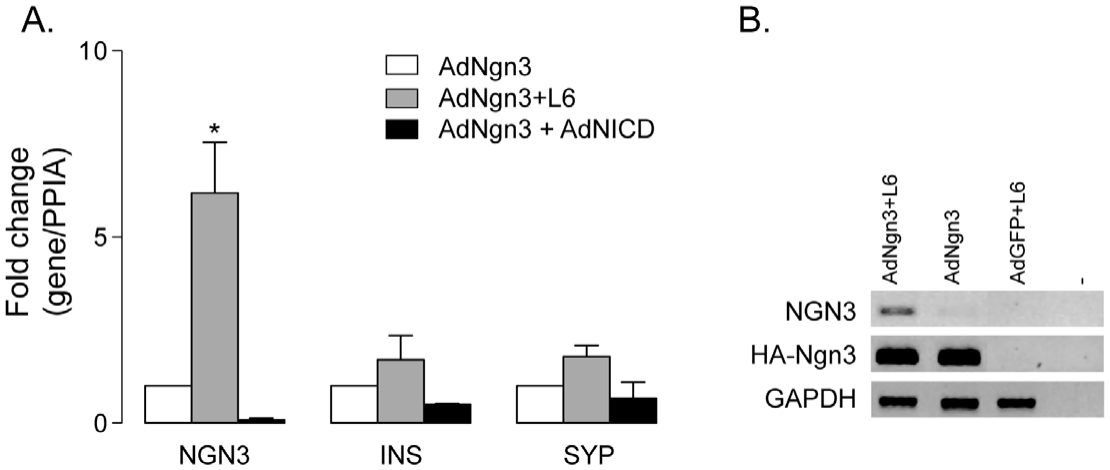
Modulation of Delta-Notch signaling affects Ngn3 induced endocrine differentiation in adult human duct cells. (A) Real time PCR quantification of endogenous Ngn3, Ins and Syp in AdGFP-Ngn3 transduced duct samples (white bars) versus gamma-secretase inhibitor L6-treated samples (gray bars), shows increased expression levels of endogenous NGN3 (*P<0.05) and the endocrine marker genes (NS, n = 3). Expression of Ngn3, Ins and Syp is reduced when AdGFP-Ngn3 cells are additionally transduced with the Notch1 intracellular domain (NICD) (black bars). Data represent mean ± SEM real-time PCR measurements of target/Ppia mRNA levels compared to AdGFP-Ngn3 transduced duct cells. (B) Conventional PCR showing an increased activation of endogenous HsNgn3 in AdGFP-Ngn3 adult human duct cells treated with L6, but no effect in control transduced duct cells. Controls of exogenous expression (HA-Ngn3 and GFP) as well as expression levels of housekeeping gene GAPDH are shown.

Ngn3 is insufficient for a full reprogramming of duct cells to a genuine endocrine phenotype. Delta-Notch signaling is candidate for permissive instruction of the Ngn3-transduced cells and therefore its role in the reprogramming process was investigated. Exogenous Ngn3 provoked a coordinated activation of genes coding for Delta-Notch signaling proteins, like Dll1, Dll4 [6] and Lnf (Table S1) that could limit reprogramming efficiency. Therefore, the impact of Notch signal inhibition was investigated by loss- and gain-of-function experiments. Addition of the gamma-secretase inhibitor L685,458 (L6) prevents the proteolytic formation of the bioactive intracellular Notch domain (NICD). L6 increased the activation of endogenous HsNgn3 in AdGFP-Ngn3 but had no effect in control transduced duct cells (Figure 4A,B). This accentuation of HsNgn3 expression, however, caused only a tendency towards increased insulin and synaptophysin mRNA levels (Figure 4A). Vice versa, when constitutively active NICD was ectopically expressed in adult human duct cells together with Ngn3, activation of endogenous HsNgn3 was blunted, leading to lower insulin and synaptophysin transcript levels (Figure 4A). Thus, active Notch signaling suppresses Ngn3 autoactivation in adult pancreatic duct cells.

### Myt1 Enhances Ngn3-induced Reprogramming

**Figure 5:**
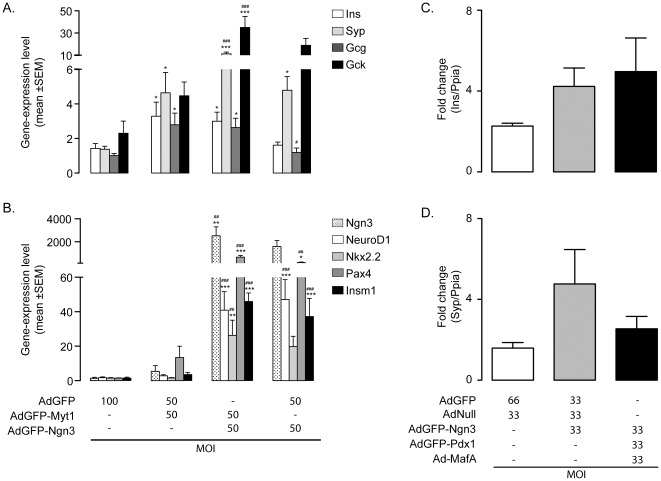
Overexpression of pancreatic endocrine transcription factors in adult human duct cells. (A, B) Effect of Myt1b overexpression in adult human duct cells. Adult human duct cells have been transduced with AdGFP, AdGFP-Ngn3 and/or AdGFP-Myt1 with a total MOI 100 and gene expression profiles have been analyzed 10 dpt. (A) Increased levels of several neuroendocrine genes are observed in AdGFP-Myt1 duct cells. The effects are enhanced by overexpression of Myt1 and Ngn3. (B) Except for Pax4, Myt1 does not upregulate known Ngn3 target genes. P<0.05 vs AdGFP (*) or AdGFP-Myt1 (#); P<0.005 vs AdGFP (**) or AdGFP-Myt1 (##); P<0.001 vs AdGFP (***) or AdGFP-Myt1 (###). (C, D) Adenoviral overexpression of Pdx1, Ngn3 and MafA in adult human duct cells does not induce the neuroendocrine genes Ins and Syp. Adult human duct cells have been transduced with control viruses AdGFP and AdNull, AdGFP-Ngn3+AdGFP+AdNull or AdGFP-Pdx1+AdGFP-Ngn3+AdMafA with a total MOI of 100 and gene expression has been analyzed 10 dpt. RT-qPCR analysis reveals that none of these conditions induced insulin or synaptophysin transcripts. Data represent mean ± SEM real-time PCR measurements of target/Ppia mRNA levels compared to non-transduced adult human duct cells (n ≥3).

Myelin transcription factor 1 (Myt1) is another important islet cell transcription factor and works in concert with Ngn3 to promote endocrine cell differentiation in the developing pancreas [11], [12], [13]. Myt1-encoding mRNA was absent from normal duct cells and although ectopic Ngn3 activated Myt1 gene expression we over-expressed Myt1b, the Myt1 isoform that is predominantly active in embryonic chicken and mouse pancreas [11], [12], to investigate whether more Myt1, alone or in combination with Ngn3 could enhance duct-to-endocrine cell reprogramming. After 10 days, Myt1b alone enhanced transcript levels of the insulin and glucagon genes to a minor extent and of the synaptophysin gene to a major extent. Only the latter one and glucokinase were increased in duct cells transduced by Ngn3 only (Figure 5A). Introduction of Myt1b in Ngn3-expressing duct cells further increased transcript levels of synaptophysin and glucokinase (P<0.05) but not of insulin and glucagon (Figure 5A). However, no significant increase in protein levels of insulin and synaptophysin could be detected. The Ngn3-induced neuro-endocrine shift occurs through recapitulation of embryonic neuro-endocrine differentiation [6], i.e. by activation of its direct target genes [6], [18], [19], [20], [21]. The abundance of transcripts coding for NeuroD1, Pax4 and Insm1 increased in duct cells transduced with Ngn3 but not Myt1 only. Myt1b further enhanced the amount of Ngn3-induced Pax4 mRNA (4-fold, P<0.001) (Figure 5B). The lack of additive effects of Myt1b on most direct targets of Ngn3 suggests that Myt1b and Ngn3 can act through parallel pathways.

### No Effect of Combined Pdx1 and MafA on Ngn3-induced Reprogramming

Adenoviral expression of Ngn3, Pdx1 and MafA has been reported to reprogram mouse pancreatic acinar cells *in vivo* into insulin-expressing cells [14]. The reprogramming potential of human duct cells either by Ngn3 alone or by combination with Pdx1 and MafA, were directly compared taking insulin and synaptophysin mRNA levels as readout. Addition of MafA and Pdx1 did not significantly enhance Ngn3-induced insulin (Figure 5C) or synaptophysin (Figure 5D) mRNA levels in duct cells.

## Discussion

The potential of reprogramming duct-to-beta cells to replenish the endocrine cell pool in diabetes patients remains controversial. Since no reliable *in situ* protein markers for human islet neogenesis are available the presence of small beta cell aggregates or single insulin expressing cells close to or in the pancreatic ducts are typically used as surrogate markers [4], [22]. Several reports described the production of endocrine material from islet-depleted leftover fractions from human pancreas after beta cell isolation [5], [8], [23], [24]. However, the efficiency of these processes is low and the newly formed endocrine cells show an inadequate response to glucose. Moreover, reports based on genetic-lineage tracing of duct cells in different mouse models of beta cell regeneration are inconclusive [25], [26], [27]. Transgenic mice in which the promoter of human carbonic anhydrase II (CAII), a ductal marker, drives the expression of (inducible) Cre recombinase showed that CAII-expressing cells can act as progenitors giving rise to new islets and acini after birth and in adult mice after partial duct ligation [25]. In contrast, when Hepatocyte nuclear factor 1b (Hnf1b), another marker of mature duct cells, was driving inducible reporter expression, fully mature duct cells did not contribute to new islet cells after partial duct ligation [27].

The fundamental question remains whether non-endocrine cells in the adult human pancreas retain a regenerative potential and whether this potential can be revealed and exploited *in vitro* using transcriptional regulators of beta cell differentiation, like Ngn3. Our prior work showed that, at least *in vitro*, human duct cells can be partly reprogrammed towards a neuro-endocrine, beta cell-like phenotype: ectopic overexpression of mouse Ngn3 activated the expression of several endocrine-specific genes, such as synaptophysin, prohormone convertase 1, glucokinase, chromogranin A [6].

A first objective of the present study was to quantify the extent of this Ngn3-mediated duct-to-endocrine cell reprogramming using genome-wide mRNA profiling. This confirmed the activation of key endocrine cell genes as previously reported [6], but at the same time provides a more realistic estimation of the overall duct-to-endocrine cell reprogramming: it turned out to be incomplete, with less than 10% of the estimated transition from primary duct to endocrine cells reached. This shift, estimated by batch analysis of the whole duct cell preparation, can underestimate the reprogramming that individual Ngn3-expressing duct cells achieve, since only 30 to 40% of cells in suspension culture were transduced, as judged by GFP expression. Moreover, it is uncertain if all transduced cells necessarily have the same reprogramming susceptibility. Although the overall transcriptome of Ngn3-transduced duct cells differed markedly from that of a typical islet endocrine cell, the phenotypic shift was highly enriched in gene clusters that are characteristic for islet endocrine cells. It also contained many genes that are more characteristic for neural tissues or with a shared abundance in islet endocrine and neural cells. It is well known that despite their different embryonic origin, endocrine islet cells and neuronal cells share similarities in their gene activity profile [28], more specifically Ngn3 expression during the development [29], [30], [31], [32], [33]. Our findings therefore suggest that the transduction of adult duct cells by Ngn3 can induce neural genes that are not normally activated in adult endocrine cells, reflecting differences between embryonic progenitor cells and the adult cells employed in the current study.

Our study on primary human cells allows a comparison of candidate Ngn3 target genes identified in earlier mouse cell line studies. A previous report [34] described 51 differentially regulated genes in the mouse pancreatic duct tumor cell line mPAC, 48 h after ectopic expression of mouse Ngn3. Only 8 of these 51 (16%) were also Ngn3-regulated in adult human duct cells: in both models Ngn3 activated endogenous Ngn3, Nkx2.2, Pax6, NeuroD1, Hes6, Isl1, somatostatin and glucokinase but not Nkx6.1 and Glut2. Despite this limited overlap, the general tissue tropism or functional ontologies of Ngn3 target genes in both species are quite similar, with many Ngn3 targets involved in neurogenesis and Notch signaling.

Mouse Ngn3 switched on the endogenous human NGN3 gene. This was surprising since Ngn3 was shown by others to inhibit its own expression during embryonic mouse development [10], [17]. This peculiar feature involved an interaction of mouse Ngn3 with the 5′ region of the human NGN3 that is at least partially modulated by Delta-Notch signaling.

A second objective was to improve reprogramming efficiency by Ngn3. We therefore first targeted the Delta-Notch pathway: since lateral inhibition by active Notch is likely to constrain endocrine specification, we suppressed it pharmacologically by gamma-secretase inhibitor. Notch suppression further accentuated the observed feed-forward stimulation of mouse on human Ngn3, but did not significantly drive endocrine reprogramming taking insulin and synaptophysin mRNA levels as markers.

In an alternative attempt to improve reprogramming, we tried to mimic combinations of transcription factors that are active in a timed cascade during in vivo endocrine differentiation. Ectopic expression of Myt1b in the developing chicken gut endoderm [11] and in mouse pancreatic progenitor cells [13] results in an increased number of glucagon-positive cells. Similar observations were reported when Ngn3 was ectopically expressed in embryonic endoderm from chicken [35] and mouse [31], [36]. A close inter-dependence of both factors is likely since Myt1-induced endocrine differentiation is blunted in embryos of Ngn3 null mice [12]. In adult human duct cells, Myt1 alone was sufficient to increase mRNA levels of insulin, glucagon and synaptophysin. Myt1 had no effect, however, on the expression of Ngn3 and several direct Ngn3 targets such as NeuroD1, Nkx2.2, and Insm1 whereas it enhanced transcription of Pax4. Myt1 thus exerts its (neuro-) endocrinogenic effects mainly independently from Ngn3 under the present experimental conditions. However, Myt1- and Ngn3-induced downstream programs seem to converge at some point since duct cells co-expressing both factors systematically showed the highest levels of synaptophysin, glucokinase and Pax4 transcripts.

The combination of MafA, Pdx1 and Ngn3 has recently been reported to drive the reprogramming of acinar to endocrine cells in the pancreas of adolescent immune-compromised mice [14]. In adult human duct cells, however, this transcription factor cocktail was not efficient as compared to Ngn3 alone. Several non-exclusive explanations are possible: perhaps differentiated acinar cells are required for proper reprogramming, potential species- or age-related factors are overlooked [37], or in the in vitro model does not support survival of crucial cell subpopulations or lacks inductive signals normally active in vivo.

These considerations are also pertinent with regard to the limited overall efficiency of Ngn3-induced reprogramming, and the limited added benefit of Notch suppression or Myt1 co-expression. In the developing pancreas, Ngn3 drives endocrine development in a complex cascade that is finely timed [1], [31] and likely topographically regulated by signals form the mesenchyme surrounding progenitor niches. None of these criteria are fulfilled in the cultured adult pancreatic duct cell preparations, with their sustained high-level expression of Ngn3. Another limitation of our model is its sole intervention at the transcriptional level with no control of epigenetic constraints. Both exocrine and endocrine cells arise from the same pancreatic progenitor and as this progenitor differentiates towards an exocrine fate, several genes important for endocrine differentiation are repressed at the epigenetic level. More work is needed to map the differences in epigenetic traits of pancreatic cell types and determine if reversal of epigenetic repressions, if feasible, can guide proper reprogramming.

In summary, ectopic expression of Ngn3 in adult human duct cells produces a focused shift of gene expression with activation of many neuron- and/or islet endocrine genes. This shift is moderately enhanced by suppression of Notch, and by co-expression of Myt1 but not MafA plus Pdx1. Though our study confirms an intriguing level of plasticity of the adult human duct cells, the overall reprogramming based on currently available protocols is limited and more is needed for differentiation to clinically useful beta cell grafts.

## Materials and Methods

### Ethics Statement

Ethical approval to use endocrine- and exocrine-enriched cells derived from donor organ was given by the Medical ethical committee of the University Hospital of the Vrije Universiteit Brussel (O.G. 016) to the Beta Cell Bank-University Hospital Brussels (permission 2010/193). Permission was also given to Dr. Heimberg by the Ethical Committee to use these cells for in vitro studies aimed to develop improved transplantation conditions (2008/048).

### Production of Recombinant Adenoviruses

Coding sequences of hemagglutinin (HA)-tagged mouse Ngn3, myc-tagged mouse Myt1b and mouse Pdx1 were subcloned in pAdTrack-CMV and constitutively expressed under the control of the CMV promoter. pAdTrack-CMV contained the enhanced green fluorescent protein (eGFP) cDNA downstream of a second CMV promoter. Recombinant, replication-deficient adenoviruses expressing GFP (AdGFP), Ngn3 in combination with GFP (AdGFP-Ngn3), Myt1 in combination with GFP (AdGFP-Myt1) and Pdx1 in combination with GFP (AdGFP-Pdx1) were generated following the standard protocol as described [38]. The recombinant adenovirus coding for MafA (AdMafA) was obtained through the Beta Cell Biology Consortium. Since this virus did not encode a reporter gene, “AdNull”, consisting of the adenoviral backbone lacking any transgene, was used as control.

### Cell isolations, Cell Culture and Viral Transduction

Human cells were obtained from heart-beating cadaveric nondiabetic donors. Full written consent was given by the family to use the donor organ for research purposes according to the Belgian laws. Ethical approval to use endocrine- and exocrine-enriched cells derived from donor organ was given by the Medical ethical committee of the University Hospital of the Vrije Universiteit Brussel. In the non-endocrine fraction, <5% expressed the islet cell marker Syp+ and >90% expressed the duct cell-specific marker CK19+ [6], [39] when cultured at least 4 days [40]. The non-endocrine cell preparation was cultured in suspension in Ham’s F10 (Bio-Whittaker, Fontenay-sous-bois, France), 0,5% BSA (Boehringer Mannheim; Indianapolis, IN), 7.5 mM glucose, 100 U/ml penicillin, 100 µg/ml streptomycin and 1 mM L-glutamine at 37°C in a humidified atmosphere. On day 4 of culture, cells were counted, transduced and further cultured in suspension at a cell density of 70 K cells/cm^2^. Alternatively, day 4 cells were counted and plated to form monolayers on tissue-culture plastic multiwells with a cell density of 70 K cells/cm^2^. Monolayer cells were cultured in the same medium as suspension with the addition of 5% FBS (Life Technologies, Carlsbad, CA, USA) and transduced 6 days later. Unless stated otherwise, the cells were maintained in monolayer culture. Transduction was performed under serum-free conditions during 4 hours at multiplicity of infection (MOI) of 50, unless stated otherwise. Under all conditions, cell culture medium was renewed every other day. The endocrine-enriched fraction consisted of 55±6% insulin+ cells, 12±4% glucagon+ cells and 22±4% non-granulated cells. The gamma secretase inhibitor L685,458 (L6) was purchased from Calbiochem (Merck, Darmstadt, Germany) and used at a final concentration of 100 nM.

### Genome-wide mRNA Expression Profiling

Quality of TRIzol (Gibco BRL, Carlsbad, CA, USA) extracted RNA was verified on a 2100 Bioanalyzer (Agilent, Waldbronn, Germany), taking minimal cutoff RIN ≥ 8. The microarray analysis was performed on 3 independent samples that each contained RNA extracted from a pool of 3 independent donor pancreata. The total number of non-selected donor organs is 9. Each RNA sample contained equal amounts of RNA extracted from the individual donor organs. A total of 5 µg of RNA was used to synthesize double-stranded cDNA with the Superscript Choice system (Invitrogen, Merelbeke, Belgium) using an oligo(dT) primer containing a T7 RNA polymerase promoter (GenSet, Paris, France). The cDNA was used as the template for an in vitro transcription reaction to synthesize biotin-labelled antisense cRNA using the BioArray high yield RNA transcript labelling kit (Enzo Diagnostics, Farmingdale, New York, USA). After fragmentation at 94°C for 35 min in fragmentation buffer (40 mM Tris, 30 mM magnesium acetate, 10 mM potassium acetate), the labelled cRNA was hybridized for 1 6 h to the Affymetrix HG133A and/or HG133B chip analyzing over 45,000 probe sets, probing expression of 39,000 transcripts corresponding to 33,000 annotated human genes (Affymetrix Inc., Santa Clara, CA, USA). Arrays were stained with phycoerythrin-streptavidin with the Affymetrix Fluidics Station 400, and scanned in the Affymetrix GeneArray2500scanner. Scanned arrays were analyzed with dChip model-based expression analysis to the array with the median intensity (AdGFP duct 3 days post transduction, intensity = 180); mean±SD intensity for the whole group was 181±39, ranging from 111 to 262 with no statistical outlying arrays [41]. Using the PM-MM model with mismatch correction, a 90% confidence interval was calculated for the fold change in gene expression in each comparison, and the lower limit of this interval (LCB) was used as a measure of differential gene expression. Transcripts were considered as differentially regulated by Ngn3 when 1.5 fold (LCB, unpaired P<0.05) up- or down-regulated in AdGFP-Ngn3 versus AdGFP controls, at 3 and/or 14 dpt. Transcripts that showed differential expression between day 3 and day 14 in AdGFP-Ngn3 duct cells but not in AdGFP control cells, were also considered Ngn3-regulated. dChip was also used for analysis of statistical over-representation of Gene Ontologies in the set of Ngn3-activated genes, taking P<0.001 as threshold for significant enrichment.

Raw data are publicly available at Gene Expression Omnibus as GSE30802 (AdGFP or AdGFP-Ngn3 transduced duct cells) and GSE30803 (untransduced human beta and duct cell preparations, and other human tissues).

### Analysis of Ngn3-induced phenotypic shift by tissue-comparative mRNA expression profiling

Abundances of AdGFP-Ngn3-regulated transcripts were visualized in the Human Genome Atlas data set [15], supplemented with our human duct and endocrine cells. After normalization to array with median intensity and modeling using mismatch correction, dChip was used to obtain a view of the global phenotypic shift by hierarchical clustering (correlation, centroid) and gene ontology clustering for statistically overrepresented cellular functions. Common genes were identified using Venny webtool [42].

### RNA Analysis

Total RNA was isolated using the RNeasy Minikit (Qiagen, Hilden, Germany). 250 ng RNA was reverse transcribed with SuperScript II Reverse Transcriptase and random hexamers according to the manufacturer’s instructions and conventional PCR was performed using Taq DNA polymerase (Invitrogen, Paisley, UK). For primer sequences, see Table S3A. Alternatively, quantitative RT-PCR was performed using TaqMan Fast Universal PCR Master Mix and TaqMan Assays (Table S3B) (Applied Biosystems, Foster City, CA, USA). As no cDNA specific assays were available for human Ngn3 (hsNgn3) nor HA-tagged mouse Ngn3 (mmNgn3), corresponding primers and probes were designed (5′–3′): hsNgn3 forward TTGCGCCGGTAGAAAGGATG, reverse GCGGACGTGGGGCAGGTC, probe CCCAGAGCCTCGGAAGACGAA; mmNgn3: forward GCTACCCATACGATGTTCCA, reverse GGAGCAGTCCCTAGGTATG, probe CCGGAGCCTCGGACCACGAA.

### Chromatin Immunoprecipitation

Chromatin immunoprecipitation experiments were performed as described [21]. Immunoprecipitation was performed with 5 µg mouse anti-HA (Roche) overnight at 4°C, followed by 4 h incubation at 4°C with 3 µg rabbit anti-mouse IgG (Sigma). For primer sequences, see Table S3C.

### Data Analysis

All values are depicted as mean ± standard error of the mean (SEM) and considered significant when P<0.05. Statistical significance of microarray data was calculated using dChip default settings, with P<0.05 corrected for multiple comparisons. All other data were statistically analyzed by 1-way ANOVA with Newman-Keuls correction.

## Supporting Information

Figure S1 **Confirmation of Ngn3-activated genes in human duct cells by conventional (A) and/or quantitative (B) PCR.** (A) Some genes are upregulated early after transduction in AdHA-Ngn3-GFP duct cells (SIM1, PAX4, NeuroD1), others only 14 days post transduction (PCSK1). The induction can be transient (SIM1) or sustain for a longer time period (PAX4). (B) Gene expression levels were compared to cyclophilin mRNA levels 14 days post transduction. Real-time PCR data confirms the observed upregulation of known Ngn3 target genes (INSM1, NEUROD1, NKX2.2, PAX4), activation of the Delta-Notch pathway (DLL1, HES6) and neuro-endocrine markers (CGA, GCK, SYP) (n≥3).
(TIF)

Figure S2 **Annotated heat map representation of Ngn3-regulated genes in suspension cultured human duct cells.** This figure represents an annotated version of Figure 1, showing the n = 140 Ngn3-activated genes on HG133A microarray. Further details on fold-regulation and P values can be found in Tables S1, S2.
(TIF)

Table S1 **Full table of Ngn3-activated genes in suspension cultured human duct cells.** 198 non-redundant transcripts are induced ≥1.5-fold (LBC, unpaired P<0.05, n = 3) at 3 and/or 14 dpt (140 transcripts on HG133A and 58 on HG133B array). * P<0.05, n = 3; right panel indicates array subtype (HG133A or B) and probe set ID. For transcripts represented on HG133A, the gene cluster (cluster A, B, C or miscellaneous, MISC) in Figure 1 is indicated in last column.
(PDF)

Table S2 **Full table of Ngn3-suppressed genes in suspension cultured human duct cells**. 83 transcripts are suppressed ≥1.5-fold (LBC, unpaired P<0.05, n = 3) at 3 and/or 14 dpt (48 transcripts on HG133A and 35 on HG133B array). *P<0.05, n = 3; right panel indicates array subtype (HG133A or B) and probe set ID.
(PDF)

Table S3 **Oligonucleotides used for** RT-PCR (A), *RT-qPCR* (B) and amplification of DNA from immunoprecipitated chromatin (C).
(PDF)
