# Supplementary figures and images for: Plasticity of Adult Human Pancreatic Duct Cells by Neurogenin3-Mediated Reprogramming
Source: PLoS One. 2012 May 14;7(5):e37055. doi: 10.1371/journal.pone.0037055 (PMC3351393; doi:10.1371/journal.pone.0037055)

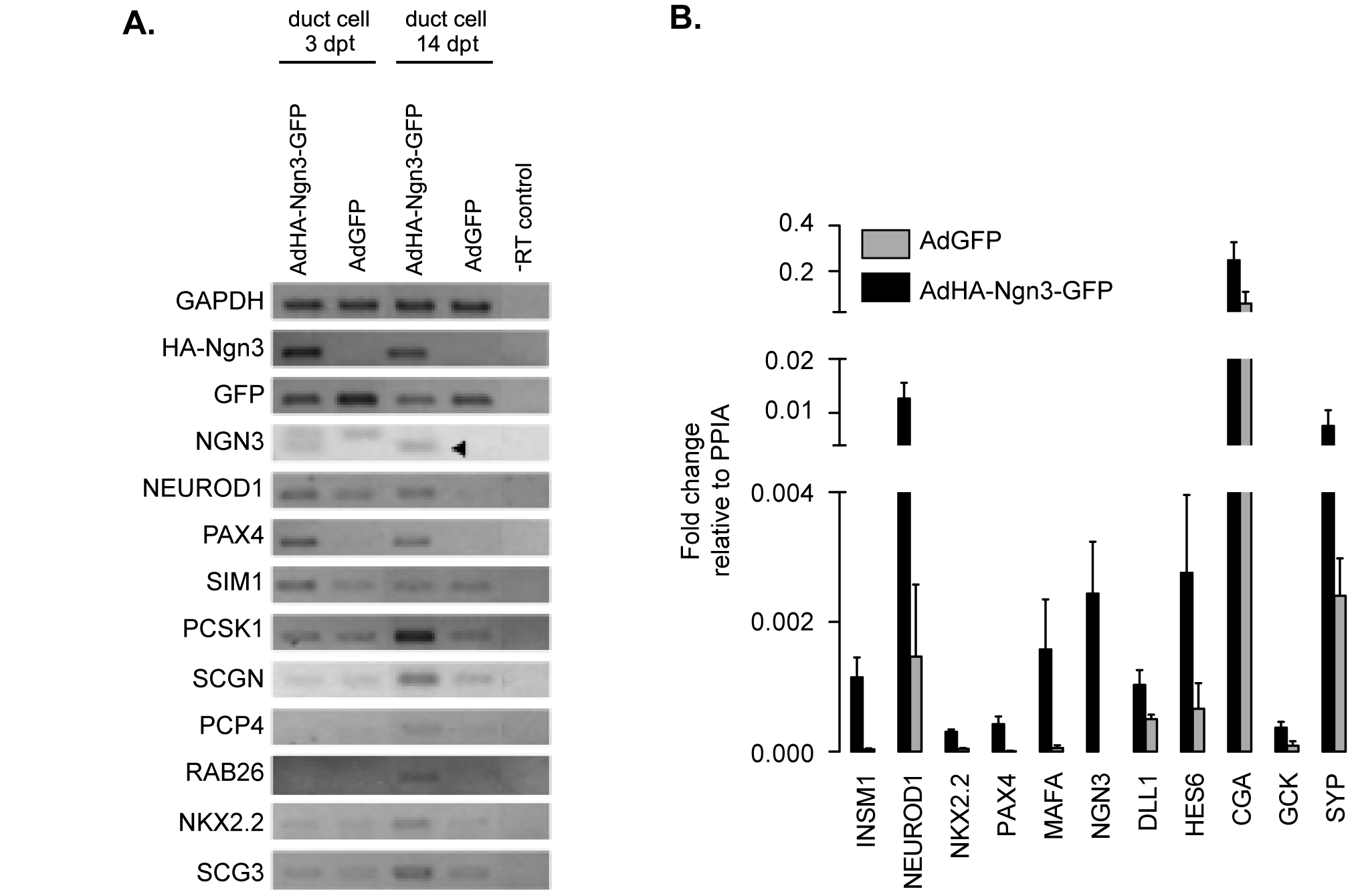

Supplement: Figure S1 — Confirmation of Ngn3-activated genes in human duct cells by conventional (A) and/or quantitative (B) PCR. (A) Some genes are upregulated early after transduction in AdHA-Ngn3-GFP duct cells (SIM1, PAX4, NeuroD1), others only 14 days post transduction (PCSK1). The induction can be transient (SIM1) or sustain for a longer time period (PAX4). (B) Gene expression levels were compared to cyclophilin mRNA levels 14 days post transduction. Real-time PCR data confirms the observed upregulation of known Ngn3 target genes (INSM1, NEUROD1, NKX2.2, PAX4), activation of the Delta-Notch pathway (DLL1, HES6) and neuro-endocrine markers (CGA, GCK, SYP) (n≥3). (TIF) [file pone.0037055.s001.tif]

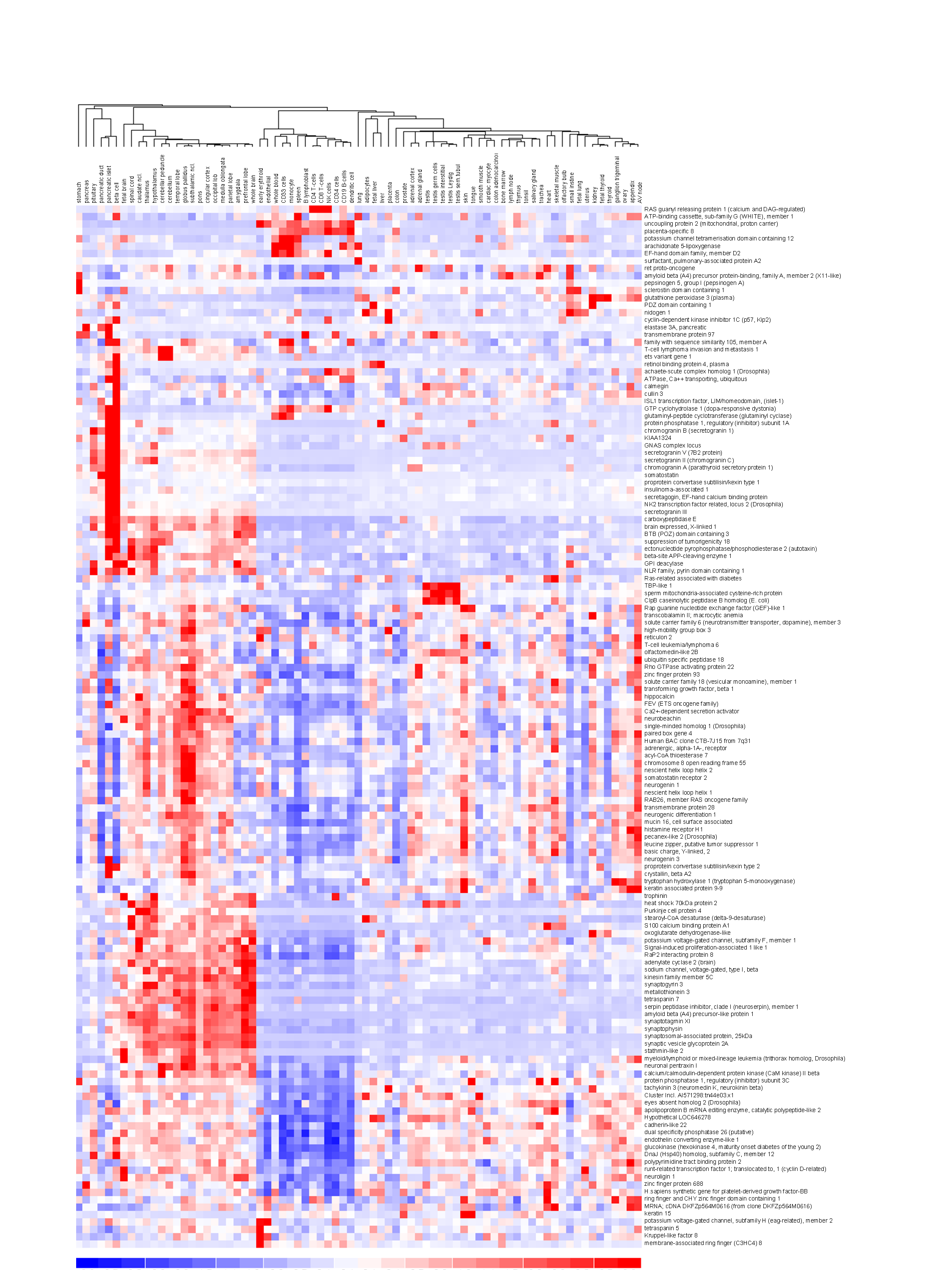

Supplement: Figure S2 — Annotated heat map representation of Ngn3-regulated genes in suspension cultured human duct cells. This figure represents an annotated version of Figure 1, showing the n = 140 Ngn3-activated genes on HG133A microarray. Further details on fold-regulation and P values can be found in Tables S1, S2. (TIF) [file pone.0037055.s002.tif]
