# Supplementary material for: Plasticity of Adult Human Pancreatic Duct Cells by Neurogenin3-Mediated Reprogramming
Source: PLoS One. 2012 May 14;7(5):e37055. doi: 10.1371/journal.pone.0037055 (PMC3351393; doi:10.1371/journal.pone.0037055)
Supplement: Table S1 — Full table of Ngn3-activated genes in suspension cultured human duct cells. 198 non-redundant transcripts are induced ≥1.5-fold (LBC, unpaired P<0.05, n = 3) at 3 and/or 14 dpt (140 transcripts on HG133A and 58 on HG133B array). * P<0.05, n = 3; right panel indicates array subtype (HG133A or B) and probe set ID. For transcripts represented on HG133A, the gene cluster (cluster A, B, C or miscellaneous, MISC) in Figure 1 is indicated in last column. (PDF) [file pone.0037055.s003.pdf]

Table S1: transcripts induced by Ad-GFP-Ngn3 in human pancreatic duct cells

Gene Symbol	Accession	Gene name	Ngn3/Control D3	Ngn3/Control D14	Ngn3 D14/D3	ARRAY HG133	Probe set ID	endocrine abundance	Cluster Fig. 2
		**Development - Neurogenesis**							
NEUROG3	NM_020999	neurogenin 3	1.7	12.5 *	5.94 *	A	207965_at	0.7	B
NEUROG1	NM_006161	neurogenin 1	2.7 *	1.1	-2.2	A	208497_x_at	0.5	B
NEUROD1	NM_002500	neurogenic differentiation 1	4.2 *	30.9 *	12.5 *	A	206282_at	21.0	B
PAX4	AB008913	paired box gene 4	1.5	2.4 *	2.01 *	A	211176_s_at	1.0	B
PAX6	AW088232	paired box gene 6 (aniridia, keratitis)	1.2	5.3 *	5.22 *	B	235795_at	-	-
NKX2-2	NM_002509	NK2 transcription factor related, locus 2 (Drosophila)	5.4 *	23.3 *	6.32 *	A	206915_at	19.9	A
Ascl1	BC001638	achaete-scute complex-like 1 (Drosophila)	1.1	48.9 *	54.9 *	A	209988_s_at	4.4	A
FOXA2	AL121722	hepatocyte nuclear factor 3, beta	2.4 *	2.6 *	-1.3	B	228004_at	-	-
EYA2	U71207	eyes absent homolog 2 (Drosophila)	2.5	2.8 *	1.79	A	209692_at	0.6	MISC
STMN2	BF967657	stathmin-like 2	1.5	5.5 *	7.35 *	A	203000_at	10.8	C
NPTX1	NM_002522	neuronal pentraxin I	45.3 *	64.1	1.33	A	204684_at	0.9	C
INSM1	NM_002196	insulinoma-associated 1	64.7 *	82.4 *	3.29 *	A	206502_s_at	25.3	A
ISL1	NM_002202	ISL1 transcription factor, LIM/homeodomain, (islet-1)	1.2	2.4 *	2.42 *	A	206104_at	26.9	A
NHLH1	M96739	nescient helix loop helix 1	1.2	2.3 *	1.85	A	214628_at	0.6	B
NHLH2	AA166895	nescient helix loop helix 2	1.6	4.3 *	3 *	A	215228_at	0.5	B
NLGN1	NM_014932	neuroligin 1	-1.5	1.0	2.09 *	A	205893_at	1.4	B
PCP4	NM_006198	Purkinje cell protein 4	2.1	4.7 *	3.4 *	A	205549_at	5.9	C
S100A1	NM_006271	S100 calcium binding protein A1	5.4 *	9.2 *	2	A	205334_at	0.5	C
SIM1	AL121948	single-minded homolog 1 (Drosophila)	1.2	1.4	1.96 *	A	206876_at	1.2	B
SERPINI1	NM_005025	serine (or cysteine) proteinase inhibitor, clade I (neuroserpin), member 1	2.0	2.6 *	-1.2	A	205352_at	2.1	C
OLFML2B	AW007573	olfactomedin-like 2B	3.6 *	2.0	-1.5	A	213125_at	0.7	B
AF1Q	BC006471	ALL1-fused gene from chromosome 1q	1.0	1.4	1.86 *	A	211071_s_at	1.2	C
PHC2	AA431100	polyhomeotic-like 2 (Drosophila)	-1.2	1.2	2 *	B	238131_at	-	-
MT3	NM_005954	metallothionein 3 (growth inhibitory factor (neurotrophic))	2.1 *	1.2	-1.6	A	205970_at	0.6	C
APBA2	AW571582	amyloid beta (A4) precursor protein-binding, family A, member 2 (X11-	2.0	3.5 *	1.05	A	209870_s_at	0.9	MISC
APLP1	U48437	amyloid beta (A4) precursor-like protein 1	1.6	3.5	5.81 *	A	209462_at	5.8	C
BACE1	NM_012104	beta-site APP-cleaving enzyme 1	1.2	1.9 *	1.54	A	217904_s_at	2.0	A
TM4SF2	NM_004615	tetraspanin 7/transmembrane 4 superfamily member 2	1.3	2.5 *	2.67 *	A	202242_at	5.0	C
TM4SF9	AF065389	tetraspanin 5/transmembrane 4 superfamily member 9	1.2	3.0 *	2.12 *	A	209890_at	1.2	MISC
GDAP1	BF002104	ganglioside-induced differentiation-associated protein 1	1.2	2.9 *	1.95	B	226269_at	-	-
		**Wnt-Notch and Nodal signaling**							
DLL1	AF196571	delta-like 1 (Drosophila)	3.7 *	1.9	-1.4	B	224215_s_at	-	-
HES6	AW249678	hairy and enhancer of split 6 (Drosophila)	5.2	16.1 *	1.91	B	226446_at	-	-
LFNG	AW151924	lunatic fringe homolog (Drosophila)	5.4 *	7.9 *	-1.2	B	228762_at	-	-
SOSTDC1	AI927000	sclerostin domain containing 1	2.2 *	45.9	-2.4	A	213456_at	1.1	MISC
NOMO2	N39536	NODAL modulator 2 (Hypothetical protein LOC283820)	41.2 *	-1.6	-1.6	A	221852_at	0.8	MISC
TGFB1	NM_000660	transforming growth factor, beta 1 (Camurati-Engelmann disease)	8.2 *	4.7	-2.3	A	203084_at	0.4	B
		**Neuroendocrine-Synaptic Function**							

CPE	NM_001873	carboxypeptidase E	1.7		3.0	*	2.16	*	A	201117_s_at	29.2	A
RTN2	NM_005619	reticulon 2	7.9	*	13.4		-1.8		A	204217_s_at	0.7	B
CADPS	NM_003716	Ca2+-dependent secretion activator	1.4		2.4	*	1.45		A	204814_at	6.2	B
ENPP2	L35594	ectonucleotide pyrophosphatase/phosphodiesterase 2 (autotaxin)	1.6		5.3	*	3.83	*	A	209392_at	13.0	A
PCSK1	NM_000439	proprotein convertase subtilisin/kexin type 1	1.4		19.7	*	15.9	*	A	205825_at	17.3	A
PCSK2	NM_002594	proprotein convertase subtilisin/kexin type 2	1.8		2.4		3.27	*	A	204870_s_at	15.4	A
RET	M31213	ret proto-oncogene	4.1	*	2.4		-3.3		A	211421_s_at	1.3	MISC
SST	NM_001048	somatostatin	-1.1		2.5		5.77	*	A	213921_at	28.4	A
TAC3	NM_013251	tachykinin 3 (neuromedin K, neurokinin beta)	1.2		34.0	*	21.7	*	A	219992_at	0.8	MISC
ECEL1	NM_004826	endothelin converting enzyme-like 1	-1.0		2.0		2.76	*	A	219914_at	1.6	MISC
CABP7	AI989906	calcium binding protein 7	4.1	*	18.1	*	3.12	*	B	243173_at	-	-
CAMK2B	AF078803	calcium/calmodulin-dependent protein kinase (CaM kinase) II beta	1.2		2.4	*	2.12	*	A	210404_x_at	1.1	C
CHGA	NM_001275	chromogranin A (parathyroid secretory protein 1)	1.4		10.4	*	12.2	*	A	204697_s_at	14.2	A
CHGB	NM_001819	chromogranin B (secretogranin 1)	1.5		2.7	*	3.13	*	A	204260_at	27.0	A
CPLX1	BC002471	complexin 1	3.7	*	3.5	*	1.11		B	223500_at	-	-
CPNE5	AB046819	copine V	-3.2		-1.3		2.62	*	B	227189_at	-	-
EFHD2	NM_024329	EF hand domain containing 2	1.2		2.3	*	1.23		A	217992_s_at	0.6	MISC
HPCA	BC001777	hippocalcin	8.1		8.8	*	7.19	*	A	205454_at	0.6	B
NBEA	NM_015678	neurobeachin	1.1		2.1	*	1.73		A	221207_s_at	1.4	B
KCNF1	AF029780	potassium voltage-gated channel, subfamily F, member 1	6.1		6.4	*	1.19		A	210263_at	0.8	C
KCNH2	NM_000238	potassium voltage-gated channel, subfamily H (eag-related), member 2	-1.0		2.4	*	2.21		A	205262_at	4.0	A
RAB26	NM_014353	RAB26, member RAS oncogene family	3.6	*	8.4	*	2.55		A	219562_at	1.0	B
RPIP8	AI197870	RaP2 interacting protein 8	-1.0		1.5		1.82	*	A	213439_x_at	1.2	C
SCGN	NM_006998	secretagogin, EF-hand calcium binding protein	3.2		23.8	*	7.59	*	A	205697_at	9.8	A
SCG2	NM_003469	secretogranin II (chromogranin C)	1.3		6.9	*	6.46	*	A	204035_at	31.1	A
SCG3	NM_013243	secretogranin III	3.9		7.5	*	5.12	*	A	219196_at	55.6	A
SGNE1	NM_003020	secretory granule, neuroendocrine protein 1 (7B2 protein)	1.1		2.8	*	2.91	*	A	203889_at	62.5	A
SCN1B	NM_001037	sodium channel, voltage-gated, type I, beta	1.5		2.5	*	1.94	*	A	205508_at	2.4	C
SV2A	NM_014849	synaptic vesicle glycoprotein 2A	1.1		1.9		2.43	*	A	203069_at	1.6	C
SYNGR3	NM_004209	synaptogyrin 3	-1.4		1.1		1.92	*	A	205691_at	1.2	C
SYP	U93305	synaptophysin	1.1		3.6	*	2.76	*	A	213200_at	1.4	C
SNAP25	NM_003081	synaptosomal-associated protein, 25kDa	-1.0		1.7		3.33	*	A	202508_s_at	14.3	C
SYT4	AB037763	synaptotagmin IV	1.0		2.8	*	2.22	*	B	223529_at	-	-
SYT11	AA626780	synaptotagmin XI	1.1		1.6		2.09	*	A	209197_at	1.6	C
SYT13	AB037848	synaptotagmin XIII	1.1		2.1	*	1.74		B	226086_at	-	-
		**Intracellular Signaling**										
PPP1R1A	NM_006741	protein phosphatase 1, regulatory (inhibitor) subunit 1A	-1.2		-1.1		1.93	*	A	205478_at	18.9	A
PPP1R3C	N26005	protein phosphatase 1, regulatory (inhibitor) subunit 3C	2.8	*	2.5	*	1.41		A	204284_at	1.1	MISC
HSPDGFBB	X63966	H.sapiens synthetic gene for platelet-derived growth factor-BB.	4.0	*	79.2		-1.8		A	217108_at	0.9	MISC
IGFBPL1	AL522781	insulin-like growth factor binding protein-like 1	2.4	*	8.1	*	3.27	*	B	227760_at	-	-
ADCY2	AU149572	adenylate cyclase 2 (brain)	1.1		2.8	*	3.62	*	A	213217_at	1.2	C
ADRA1A	D32202	adrenergic, alpha-1A-, receptor	15.2	*	4.7		-3.3		A	211491_at	0.5	B
GNAS	AA401492	GNAS complex locus	1.9		5.0	*	2.1	*	A	214157_at	9.5	A
HRH1	NM_000861	histamine receptor H1	3.3	*	4.6		1		A	205579_at	0.4	B

GNG2	AK026424	guanine nucleotide binding protein (G protein), gamma 2	1.2		10.5	*	9.75	*	B	224964_s_at	-	-
SSTR2	BC000256	somatostatin receptor 2	2.1		4.9	*	2.54	*	A	214597_at	0.4	B
RAPGEFL1	NM_016339	Rap guanine nucleotide exchange factor (GEF)-like 1	1.8		2.4	*	1.25		A	218657_at	0.9	MISC
RASGRP1	NM_005739	RAS guanyl releasing protein 1 (calcium and DAG-regulated)	1.4		2.1	*	1.77		A	205590_at	3.1	MISC
RRAD	NM_004165	Ras-related associated with diabetes	4.0	*	1.0		-4.3		A	204802_at		A
MAP3K4	AI633559	mitogen-activated protein kinase kinase kinase 4	1.5		4.5	*	1.7		B	238623_at	-	-
PLCXD3	AL133721	phosphatidylinositol-specific phospholipase C, X domain containing 3	-1.9		5.8	*	5.17		B	239270_at	-	-
PRKCE	AI247134	Protein kinase C, epsilon -grade E annotation	3.0	*	1.3		-2.5		B	239011_at	-	-
TNK2	BE676245	activated p21cdc42Hs kinase	4.8	*	1.8		-2.5		B	228279_s_at	-	-
TIAM1	U90902	T-cell lymphoma invasion and metastasis 1	-1.2		1.5		2.03	*	A	213135_at	2.5	A
		**Nuclear - Transcription**										
CUL3	NM_003590	cullin 3	1.0		7.3	*	6.87	*	A	201372_s_at	3.6	A
CDKN1C	R78668	cyclin-dependent kinase inhibitor 1C (p57, Kip2)	2.2	*	2.0		1.78		A	213182_x_at	1.3	MISC
NALP1	NM_021730	NACHT, leucine rich repeat and PYD containing 1	1.1		10.3	*	9.86	*	A	218380_at	7.0	A
HMGB3	NM_005342	high-mobility group box 3	1.5		2.1	*	1.42		A	203744_at	0.9	B
SOX2	AI669815	SRY (sex determining region Y)-box 2	2.3		15.1	*	10.4	*	B	228038_at	-	-
BPY2	NM_004678	basic charge, Y-linked, 2	4.0	*	1.8		-2.2		A	208331_at	0.5	B
BEX1	NM_018476	brain expressed, X-linked 1	-1.2		1.6		2.15	*	A	218332_at	7.4	A
LOC51337	NM_016647	mesenchymal stem cell protein DSCD75	1.1		2.1	*	1.75		A	218500_at	0.7	B
KCTD12	AI718937	potassium channel tetramerisation domain containing 12	1.2		2.1	*	1.92	*	A	212192_at	4.1	MISC
BTBD3	NM_014962	BTB (POZ) domain containing 3	1.0		2.0	*	2	*	A	202946_s_at	7.5	A
ST18	NM_014682	suppression of tumorigenicity 18 (breast carcinoma) (zinc finger protein	1.4		7.9	*	4.94	*	A	206135_at	11.4	A
ZNF505	AC007204	zinc finger protein 505	2.9	*	1.4		-1.8		A	215758_x_at	1.1	B
RUNX1T1	NM_004349	runt-related transcription factor 1; translocated to, 1 (cyclin D-related)	1.3		3.0	*	2.17	*	A	205529_s_at	1.7	MISC
FEV	NM_017521	FEV (ETS oncogene family)	-1.0		9.3	*	12.7	*	A	207260_at	0.8	B
KLF8	NM_007250	Kruppel-like factor 8	2.3	*	-1.6		-2.1		A	219930_at	1.1	MISC
TBPL1	NM_004865	TBP-like 1	1.4		1.8	*	-1		A	208398_s_at	0.8	MISC
HIC2	AK024950	Hypermethylated in cancer 2	-1.1		1.5		3.78	*	B	233374_at	-	-
ETV1	BE881590	ets variant gene 1	-1.5		1.6		2.2	*	A	221911_at	9.6	A
		**RNA and protein turnover - Cellular response to stress**										
EXOSC4	AI571298	exosome component 4	7.0	*	5.1	*	-1.4		A	91682_at	0.8	MISC
APOBEC2	NM_006789	apolipoprotein B mRNA editing enzyme, catalytic polypeptide-like 2	2.3	*	1.6		-1		A	206160_at	1.4	MISC
MIR	AA770170	c-mir/membrane-associated ring finger (C3HC4) 8 (MARCH8)	1.2		2.0	*	1.75		A	221824_s_at	0.8	MISC
ERO1LB	AL045717	endoplasmic oxidoreductin 1-like protein b	1.8		2.9	*	1.55		B	231944_at	-	-
PTBP2	NM_021190	polypyrimidine tract binding protein 2	1.7		2.4	*	1.45		A	218683_at	0.7	MISC
USP18	NM_017414	ubiquitin specific protease 18	2.3		7.8	*	1.37		A	219211_at	0.8	B
RCHY1	AV694093	ring finger and CHY zinc finger domain containing 1	-3.4		1.1		4.03	*	A	212743_at	1.1	MISC
MSI2	BF435123	musashi homolog 2 (Drosophila)	2.3	*	1.1		-1.3		B	225238_at	-	-
IBRDC2	BE274992	IBR domain containing 2	1.0		3.6	*	1.84		B	239704_at	-	-
DNAJC12	AF176013	DnaJ (Hsp40) homolog, subfamily C, member 12	1.9		8.7	*	-1.3		B	223721_s_at	-	-
CLGN	NM_004362	calmegin	1.0		2.7	*	1.98		A	205830_at	4.2	A
HSPA2	U56725	heat shock 70kDa protein 2	1.7		2.2	*	1.92	*	A	211538_s_at	1.1	MISC
GPX3	NM_002084	glutathione peroxidase 3 (plasma)	-1.3		1.1		1.81	*	A	201348_at	1.3	MISC

SKD3	AI655698	suppressor of potassium transport defect 3	2.2	*	1.0		-1.9		A	221845_s_at	0.8	MISC
		**Metabolic Phenotype**										
SLC6A3	NM_001044	solute carrier family 6 (neurotransmitter transporter, dopamine), membe	1.0		2.2	*	1.92		A	206836_at	0.9	B
SLC18A1	NM_003053	solute carrier family 18 (vesicular monoamine), member 1	1.2		2.4	*	2.54	*	A	207074_s_at	0.7	B
QPCT	NM_012413	glutaminyl-peptide cyclotransferase (glutaminyl cyclase)	1.1		2.1	*	2.04	*	A	205174_s_at	28.0	A
GCH1	NM_000161	GTP cyclohydrolase 1 (dopa-responsive dystonia)	1.0		4.2	*	3.36	*	A	204224_s_at	20.3	A
PGAP1	AV705244	GPI deacylase	1.2		1.9	*	1.54		A	213469_at	4.4	A
TPH1	AI350339	tryptophan hydroxylase 1 (tryptophan 5-monooxygenase)	2.5		191.4	*	43	*	A	214601_at	1.0	B
GCK	M69051	glucokinase (hexokinase 4, maturity onset diabetes of the young 2)	1.4		1.9		1.94	*	A	211167_s_at	1.7	MISC
UCP2	U94592	uncoupling protein 2 (mitochondrial, proton carrier)	2.8	*	6.0		3.56		A	208998_at	0.8	MISC
RBP4	NM_006744	retinol binding protein 4, plasma	1.0		1.8		2.78	*	A	219140_s_at	48.0	A
ATP2A3	AF068220	ATPase, Ca++ transporting, ubiquitous	5.7	*	2.3		-2.3		A	207521_s_at	2.0	A
BACH	AL031848	brain acyl-CoA hydrolase	2.0	*	1.1		-2.1		A	215728_s_at	0.5	B
SCD	AB032261	stearoyl-CoA desaturase (delta-9-desaturase)	-1.2		1.6		2.4	*	A	200832_s_at	5.5	C
OGDHL	NM_018245	oxoglutarate dehydrogenase-like	-1.3		-1.3		2.12	*	A	219277_s_at	1.6	C
LOC402295	AC003989	Similar to Argininosuccinate synthase (Citrulline--aspartate ligase)	4.0	*	2.1		-1.4		A	216564_at	0.7	B
TCN2	NM_000355	transcobalamin II; macrocytic anemia	1.9	*	1.4		-1.1		A	204043_at	0.7	B
ALOX5	NM_000698	arachidonate 5-lipoxygenase	1.4		2.3	*	-1.2		A	204446_s_at	5.2	MISC
ABCG1	NM_004915	ATP-binding cassette, sub-family G (WHITE), member 1	1.0		1.1		2	*	A	204567_s_at	2.2	MISC
SCARB1	AF131830	Scavenger receptor class B, member 1 = CD36 antigen - like1	-1.5		1.2		2.23	*	B	233994_at	-	-
PDZK1	NM_002614	PDZ domain containing 1	2.3	*	1.9		1.09		A	205380_at	1.0	MISC
ELA3A	D00306	elastase 3A, pancreatic (protease E)	1.9	*	-1.6		-4.7		A	210080_x_at	2.1	A
PGA5	AI570199	pepsinogen 5, group I (pepsinogen A)	10.6	*	5.4		-1.8		A	213265_at	2.7	A
		**Structural - Extracellular Matrix**										
SPTBN4	AF311856	spectrin, beta, non-erythrocytic 4	-1.6		1.1		2.36	*	B	224144_at	-	-
PCDHB10	AF131761	protocadherin beta 10	10.0		3.6	*	2.61		B	223854_at	-	-
MCSP	NM_030663	mitochondrial capsule selenoprotein	2.1	*	1.8		-1.4		A	206406_at	1.1	MISC
NID	BF940043	nidogen (enactin)	1.1		2.1		3.38	*	A	202007_at	0.9	MISC
CDH22	AF035300	cadherin-like 22	2.6	*	1.3		-1		A	215181_at	1.1	MISC
CRYBA2	NM_005209	crystallin, beta A2	1.0		12.1	*	14.7	*	A	220136_s_at	1.2	B
KRT15	NM_002275	keratin 15	2.7		24.0	*	-1.5		A	204734_at	1.4	MISC
NYX	Z93015	nyctalopin	45.1	*	17.8		-3.2		B	234496_x_at	-	-
MUC16	NM_024690	mucin 16	4.9	*	1.7		-3		A	220196_at	0.7	B
KRTAP9-9	NM_030975	keratin associated protein 9-9	6.5	*	6.9		1.27		A	220972_s_at	0.6	B
SFTPA2	NM_006926	surfactant, pulmonary-associated protein A2	5.1	*	8.9		1.13		A	218835_at	1.5	MISC
KRTAP4-10	AJ406942	keratin associated protein 4-10	3.1	*	2.2		-1.6		B	234635_at	-	-
KRTAP9-4	AJ406948	keratin associated protein 9-2 /// keratin associated protein 9-3	3.5	*	4.8	*	1.41		B	233640_x_at	-	-
TRO	AF349719	trophinin	1.1		1.4		2.35	*	A	211700_s_at	0.8	MISC
		**Unassigned Function - EST**										
KIAA1324	AB037745	maba1	1.7		3.0	*	1.65		A	221874_at	6.8	A
TMEM28	NM_015686	transmembrane protein 28	2.1	*	3.1		1.26		A	206299_at	0.8	B
ARHGAP22	NM_021226	Rho GTPase activating protein 22	1.6		4.0	*	2.17		A	206298_at	0.7	B

TCL6	AF195820	T-cell leukemia/lymphoma 6	1.3		2.8		2.09	*	A	219840_s_at	0.5	B
CD99L2	AL136580	CD99 antigen-like 2	1.8		2.0	*	1.17		B	223041_at	-	-
SUSD2	AK026431	sushi domain containing 2	87.4	*	69.0	*	-1.3		B	227480_at	-	-
RPESP	AW451999	RPE-spondin	-1.0		1.1		2.4	*	B	230661_at	-	-
ECRG4	AF325503	esophageal cancer related gene 4 protein	-1.2		2.1		2.84	*	B	223623_at	-	-
PLAC8	NM_016619	placenta-specific 8	1.3		2.8	*	2.04	*	A	219014_at	1.8	MISC
MGC1136	NM_024025	dual specificity phosphatase 26 (putative)/hypothetical protein MGC113	-1.1		1.5		2.92	*	A	219144_at	1.1	MISC
PCNXL2	NM_024938	pecanex-like 2 (Drosophila) (hypothetical protein FLJ11383)	5.7	*	4.1		-2.3		A	220461_at	0.4	B
SDAD1	AA768015	SDA1 domain containing 1	-5.3		1.0		7.6	*	B	242190_at	-	-
SIPA1L1	AB007900	Signal-induced proliferation-associated 1 like 1	-1.0		1.2		1.82	*	A	202254_at	1.1	C
-	AW188087	CDNA FLJ30428 fis, clone BRACE2008941	1.3		2.3	*	1.65		B	226809_at	-	-
-	AV723984	CDNA FLJ45905 fis, clone OCBBF3026576	1.4		2.3	*	1.92		B	230403_at	-	-
-	N30138	chromosome 14 open reading frame 31	2.0	*	1.5		-1.6		B	225464_at	-	-
-	AB058892	chromosome 19 open reading frame 30	1.0		4.0	*	4.75	*	B	223913_s_at	-	-
-	AW452080	chromosome 9 open reading frame 47	1.1		-1.2		2.31	*	B	231286_at	-	-
-	AF131798	Clone 25119 mRNA sequence	1.4		3.4	*	2.25		B	233310_at	-	-
-	AL080072	DKFZp564M0616 (from clone DKFZp564M0616)	2.2	*	-1.9		2.84		A	215763_at		
-	AA682853	EST	3.6	*	1.9		-1.6		B	243950_at	-	-
-	AA825243	EST	3.7	*	1.4		-1.8		B	243950_at	-	-
-	AI912225	EST	4.8	*	1.7		-1.9		B	243950_at	-	-
-	AI963605	EST	3.6	*	1.5		-1.1		B	243950_at	-	-
-	AL040051	EST	2.4	*	1.6		-1.9		B	243950_at	-	-
-	AV685992	EST	-1.1		2.0		2.45	*	B	243950_at	-	-
-	AW590038	EST	2.5	*	1.3		-1.7		B	243950_at	-	-
-	AK026856	FLJ23203 fis, clone ADKA02487	-1.1		2.0		3.31	*	A	216794_at	0.9	MISC
-	BC001200	hypothetical protein BC009862	1.4		2.9	*	1.99		B	232642_at	-	-
-	NM_019018	hypothetical protein FLJ11127	1.5		2.8	*	1.85		A	219694_at	2.3	A
-	BF002149	hypothetical protein FLJ20245	6.4	*	2.5		-1.9		B	229708_at	-	-
-	AU150619	hypothetical protein LOC149351	-1.0		1.1		4.27	*	B	232450_at	-	-
-	AI766925	hypothetical protein LOC199964	3.4	*	2.1		-1		B	230822_at	-	-
-	AA600175	hypothetical protein LOC253012	8.0	*	34.5	*	7.55	*	B	242601_at	-	-
-	BG396614	hypothetical protein LOC283683	2.0		2.9	*	1.49		A	217520_x_at	1.3	MISC
-	AU151211	hypothetical protein LOC284723	12.6	*	27.0	*	1.64		B	232245_at	-	-
-	BF038366	hypothetical protein MAC30	-1.1		2.2	*	1.47		A	212281_s_at	1.2	A
-	AA708152	hypothetical protein MGC23911	2.9	*	1.9		-2		B	236430_at	-	-
-	AV762108	hypothetical protein MGC2477	1.2		2.5	*	2.39	*	B	238680_at	-	-
-	AB051499	KIAA1712	1.4		2.2	*	1.41		B	231850_x_at	-	-
-	AI350500	similar to hypothetical protein MGC13138	3.3	*	1.6		-1.8		A	213529_at	0.9	MISC
-	AI377780	Similar to hypothetical protein MGC39725	-1.6		1.3		3.36	*	B	231525_at	-	-
