# Supplementary material for: Plasticity of Adult Human Pancreatic Duct Cells by Neurogenin3-Mediated Reprogramming
Source: PLoS One. 2012 May 14;7(5):e37055. doi: 10.1371/journal.pone.0037055 (PMC3351393; doi:10.1371/journal.pone.0037055)
Supplement: Table S2 — Full table of Ngn3-suppressed genes in suspension cultured human duct cells. 83 transcripts are suppressed ≥1.5-fold (LBC, unpaired P<0.05, n = 3) at 3 and/or 14 dpt (48 transcripts on HG133A and 35 on HG133B array). *P<0.05, n = 3; right panel indicates array subtype (HG133A or B) and probe set ID. (PDF) [file pone.0037055.s004.pdf]

Table S2: Gene expression suppressed by Ad-GFP-ngn3 in human pancreatic duct cells

Gene Symbo	Accession	Gene name	Ngn3/Control D3		Ngn3/Control D14		Ngn3 D14/D3		ARRAY HG133	Probe set ID
		**Pancreatic Non-Endocrine**								
CTRB1	NM_001906	chymotrypsinogen B1	1.0		-2.3		-4.1	*	A	205971_s_at
ELA3A	BC005918	elastase 3A, pancreatic (protease E)	1.8		-1.6		-4.6	*	A	211738_x_at
ELA3B	NM_007352	elastase 3B	1.9		-1.7		-6.9	*	A	206151_x_at
KLK12	AF135025	kallikrein 12	1.1		-1.3		-4.5	*	B	234316_x_at
MP1	NM_002421	matrix metalloproteinase 1 (interstitial collagenase)	-1.9	*	1.0		-1.2		A	204475_at
PRSS2	NM_002770	protease, serine, 2 (trypsin 2)	1.0		-2.0		-3.8	*	A	205402_x_at
REG1A	AF172331	regenerating islet-derived 1 alpha	-1.1		-1.8		-1.9	*	A	209752_at
REG1B	NM_006507	regenerating islet-derived 1 beta	-1.1		-2.3		-3.0	*	A	205886_at
REG3A	NM_002580	regenerating islet-derived 3 alpha (pancreatitis-associated protein)	-1.0		-3.0		-4.4	*	A	205815_at
REGL	NM_006508	regenerating islet-derived-like, pancreatic stone protein-like	-1.1		-2.3	*	-4.4	*	A	207778_at
RNASE1	NM_002933	ribonuclease, RNase A family, 1 (pancreatic)	-1.2		-1.8		-2.6	*	A	201785_at
SPINK1	NM_003122	serine protease inhibitor, Kazal type 1	-1.0		-1.6		-1.8	*	A	206239_s_at
PRSS3	U66061	protease, serine, 3 (mesotrypsin)	-1.1		-2.4		-4.2	*	A	215395_x_at
ALB	X57348	albumin	-1.2		-4.2	*	-3.8		A	211298_s_at
		**Metabolic Phenotype**								
NQ01	AI039874	NAD(P)H dehydrogenase, quinone 1	-2.0	*	-1.4		1.1		A	201467_s_at
CYP2C8	NM_030878	cytochrome P450, family 2, subfamily C, polypeptide 8	-1.9		-3.1	*	1.2		A	208147_s_at
APOL2	NM_030882	apolipoprotein L, 2	-1.8		-2.9	*	1.0		A	221013_s_at
AKR1B10	NM_020299	aldo-keto reductase family 1, member B10 (aldose reductase)	-2.2	*	-1.0		1.2		A	206561_s_at
FABP5	NM_001444	fatty acid binding protein 5 (psoriasis-associated)	-2.0	*	1.6		1.5		A	202345_s_at
CSE-C	AF300796	cytosolic sialic acid 9-O-acetylesterase homolog	-394.3	*	1.0		1.0		B	223744_s_at
PPGB	NM_000308	protective protein for beta-galactosidase (galactosialidosis)	-1.3		-1.9	*	1.0		A	200661_at
ATP2B3	AW615612	ATPase, Ca++ transporting, plasma membrane 3	-424.5	*	-1.5		660.1		A	215911_x_at
		**Development - Transcription - DNA interacting**								
S100P	NM_005980	S100 calcium binding protein P	-1.9		-2.8	*	-1.1		A	204351_at
HOXD9	NM_014213	homeo box D9 (HOXD9)	-4.1	*	-2.6		-2.9		A	205605_at
PLXNA1	AL162013	plexin A1	-1.6		-3.2	*	-1.5		A	214781_at
PX19	AF112203	px19-like protein	1.2		1.1		-2.1	*	B	224232_s_at
OTX1	AI813505	orthodenticle homolog 1 (Drosophila)	-2.7		-95.2	*	-70.6		B	238839_at
ATF3	NM_001674	activating transcription factor 3	-1.2		-1.4		-2.0	*	A	202672_s_at
WBSCR20B	AI768378	Williams-Beuren Syndrome critical region protein 20 copy B	-2.3	*	-2.1	*	1.3		A	213670_x_at
SFN	X57348	stratifin	-2.1	*	-1.7		-1.2		A	33323_r_at
NOL6	AK026258	nucleolar protein family 6 (RNA-associated)	-2.6	*	-1.4		-1.6		B	232566_at
BRF1	N91109	BRF1 homolog, subunit of RNA polymerase III transcription initiation factor IIIB (S. cerevisiae)	-2.9		-3.9	*	-3.4	*	A	215677_s_at
PRDM9	NM_020227	PR domain containing 9	-1.1		-2.4	*	-4.3	*	A	221151_at
CHRAC1	AK023537	chromatin accessibility complex 1	-1.0		-1.4		-3.4	*	B	231764_at
MYC	NM_002467	v-myc myelocytomatosis viral oncogene homolog (avian)	-1.8	*	-1.2		-1.5		A	202431_s_at
		**Signaling**								
PRRPC	NM_002838	protein tyrosine phosphatase, receptor type, C	-2.3	*	-4.9		-6.2		A	207238_s_at
FGFR2	AB030075	fibroblast growth factor receptor 2	-2.3	*	-2.6		-1.7		A	211398_at
MAP3K5	NM_005923	mitogen-activated protein kinase kinase kinase 5	1.2		-1.1		-2.1	*	A	203837_at
DUSP4	BC002671	dual specificity phosphatase 4	-1.0		-1.3		-2.3	*	A	204015_s_at
EDG6	NM_003775	endothelial differentiation, G-protein-coupled receptor 6	-2.3		-3.2	*	1.0		A	206437_at

PTGER1	AI480353	prostaglandin E receptor 1 (subtype EP1), 42kD	1.0		-1.1		-3.6	*	B	231201_at
		**Immological**								
IGLC2	H53689	Immunoglobulin lambda variable 3-21	-212.8	*	-110.9		1.0		A	215214_at
TSCOT	AF242557	thymic stromal co-transporter	-2.5		-63.1	*	-87.4	*	B	223816_at
NCF1	AW072388	neutrophil cytosolic factor 1 (47kDa, chronic granulomatous disease, autosomal 1)	1.2		2.3		-8.9	*	A	214084_x_at
		**Response to stress-Protein turnover**								
HSPA6	NM_002155	heat shock 70kDa protein 6 (HSP70B')	-1.2		-1.4		-2.1	*	A	213418_at
SQSTM1	N30649	sequestosome 1	-2.1	*	-1.9		-2.0		A	213112_s_at
MDN1	AL096678	MDN1, midasin homolog (yeast)	-2.6	*	-1.1		2.1		B	234854_at
CFLAR	AA971429	CASP8 and FADD-like apoptosis regulator	-2.9	*	-3.5		-3.5		B	237367_x_at
		**Structural**								
MUC1	AI610869	mucin 1, transmembrane	-1.7	*	-1.7		-1.5		A	213693_s_at
KLHL1	AF126749	kelch-like 1 (Drosophila)	-3.3	*	-2.4		-1.9		A	216391_s_at
BLOC1S3	BF434644	Biogenesis of lysosome-related organelles complex-1, subunit 3 (elastin family)	-1.3		-2.4	*	-5.6		B	230576_at
KATNB1	AI659998	katanin p80 (WD40-containing) subunit B 1	-4.1	*	-7.4		-8.8		B	229077_at
UPK1B	NM_006952	uroplakin 1B (tetraspanin family)	-1.7		-1.8	*	-1.1		A	210064_s_at
		**Unknown Function - EST**								
RARRES2	BC000069	retinoic acid receptor responder (tazarotene induced) 2	-1.1		-1.5		-2.2	*	A	209496_at
CDRT1	U43383	CMT1A duplicated region transcript 1	-2.6		-10.2	*	-1.9		A	215999_at
LOC388503	AV700829	complement C3 protein (GPC3) precursor-like	-64.1	*	-4.6		10.7		B	231691_at
SDBCAG84	AF308298	serologically defined breast cancer antigen 84	-6.3	*	2.4		2.7		A	215257_at
LOC201140	AI741629	Similar to DKFZP566O084 protein	-1.8		-3.3	*	-3.3	*	B	230915_at
C14orf151	BC006173	chromosome 14 open reading frame 151 /// chromosome 14 open reading frame 151	1.4		1.4		-2.4	*	B	224469_s_at
-	U82695	CDNA FLJ25090 fis, clone CBL08887	-1.0		-1.0		-3.8	*	B	233116_at
-	NM_020356	chromosome 20 open reading frame 32	2.0		-5.2	*	-3.5		A	220888_s_at
-	BC003550	chromosome 7 open reading frame 20	1.1		-1.1		-1.9	*	B	223811_s_at
-	BF339357	chromosome 9 open reading frame 9	-2.7	*	-2.0		-2.2		B	239222_at
-	AA601997	EST	-2.9	*	-1.2		-1.5		B	237154_at
-	AI002125	EST	1.5		-42.0		-105.8	*	B	241204_at
-	AI040777	EST	-1.5		-2.4	*	-2.3	*	B	241334_at
-	AI492953	EST	-3.2	*	-8.0		-7.8		B	239696_at
-	AI827936	EST	-3.1	*	-17.0		-44.1		B	243925_at
-	AI973099	EST	-704.0	*	1.0		1.0		B	242264_at
-	AL040051	EST	2.4		1.6		-1.9	*	B	225481_at
-	AL537303	EST	-1.0		-1.5		-2.6	*	B	230600_at
-	AW977708	EST	-2.4	*	-1.4		-1.2		B	238899_at
-	BE465909	EST	-2.2	*	-1.3		-1.1		B	239662_x_at
-	BE645588	EST	-2.8	*	-1.3		1.1		B	243429_at
-	BF055302	EST	-1.2		-1.3		-2.7	*	B	237547_at
-	BF512216	EST	-2.8	*	-1.1		-1.2		B	230111_at
-	Y12839	hypothetical protein BC014602	-2.3	*	-2.1		-1.8		B	234498_at
-	NM_018331	hypothetical protein FLJ11125	-2.3	*	-1.7		-1.6		A	219776_s_at
-	AU157605	hypothetical protein FLJ11236	-2.3	*	-1.3		-1.6		B	228433_at
-	NM_024765	hypothetical protein FLJ12649	-1.9	*	-1.6		-1.4		A	219576_at
-	AA007367	hypothetical protein FLJ32356	1.2		1.1		-2.6	*	B	229690_at
-	AL137364	hypothetical protein MGC24039	-2.1	*	1.3		1.0		B	228551_at
-	NM_018614	hypothetical protein PRO2012	-1.1		1.0		-64.4	*	A	220883_at
