# Supplementary material for: Plasticity of Adult Human Pancreatic Duct Cells by Neurogenin3-Mediated Reprogramming
Source: PLoS One. 2012 May 14;7(5):e37055. doi: 10.1371/journal.pone.0037055 (PMC3351393; doi:10.1371/journal.pone.0037055)
Supplement: Table S3 — Oligonucleotides used for RT-PCR (A), RT-qPCR (B) and amplification of DNA from immunoprecipitated chromatin (C). (PDF) [file pone.0037055.s005.pdf]

A.

Template	Forward (5'-3')	Reverse (5'-3')	cDNA PCR product (bp)
Gapdh	ACCACAGTCCATGCCATCAC	TCCACCACCCTGTTGCTGTA	452
GFP	GTCCAGGAGCGCACCATCT	GTTCTTCTGCTTGTCGGCCA	200
HA-Ngn3	GCTACCCATACGATGTTCCA	CAGTCACCCACTTCTGCTTC	193
hsNgn3	CCACGGCCCTCGCTGCTC	GCGGACGTGGGGCAGGTC	155
NeuroD1	AGGCCCCAGGGTTATGAGAC	TGAGTCCTCCTCTGCGTTCA	218
Nkx2.2	GCACCGAGGGCCTTCAGTA	CCGGGGTCTCCTTGTCATT	123
Pax4	CGGCTAGCAGTCAGTGGAAT	GCAGGACTCGGTTGATGGA	292
Pcp4	CAACCAATGGAAAAGACA	CCAGCCTTCTTCTTCTG	151
Pcsk1	GGGTGGAGAAGATGGTGGAT	CGCAGGGTAAGGAAGAAGCA	460
Rab26	CAGGAAGAAGACCCCCAAGA	TTTGTTCCGGAAGTCAATGC	307
Scg3	CAACCCAGGAGGAAAGACAG	CCTCGGCTTCTTCCTTGT	203
Scgn	CAGAAGTGGATGGGTTTGTC	GTTGATTTTCAGCCCAAGAC	167
Sim1	AGAAGCCTATGAAAACAGCA	CATTAATGGAAGCCAGTTG	272

B.

Template	Assay ID
Dll1	Hs00194509_m1
Gcg	Hs00174967_m1
Gck	Hs00277220_m1
Hes1	Hs00172878_m1
Ins	Hs00355773_m1
Insm1	Hs00357871_s1
MafA	Hs01651425_s1
NeuroD1	Hs00159598_m1
Nkx2.2	Hs00159616_m1
Pax4	Hs00173014_m1
Ppia	Hs99999904_m1
Syp	Hs00300531_m1

C.

Template	Forward (5'-3')	Reverse (5'-3')	genomic PCR product (bp)
Ngn3	TGGCCCGGGCGAAGCAGATA	GCCCCAGCCCCAAAGGAGAAAA	117
NeuroD1	CCCTCTATCCCCGTCCCTTCTG	CCACGTGACCTGCCCATTTGTA	162
BRCA-1	GAGTCCTAGCCCTTTCACCCATAC	GTGATGTTCCTGAGATGCCTTTG	288
CTLA-4	ACAAGGCTCAGCTGAACCTGGGT	CTGCCGCCCAACTGCTCC	259
